# Age-associated epigenetic modifications in human DNA increase its immunogenicity

**DOI:** 10.18632/aging.100121

**Published:** 2010-01-27

**Authors:** Anshu Agrawal, Jia Tay, Gi-Eun Yang, Sudhanshu Agrawal, Sudhir Gupta

**Affiliations:** Division of Basic and Clinical Immunology, Med. Sci. I C-240A, University of California, Irvine CA 92697, USA

**Keywords:** Dendritic cells, aging, self DNA, Methylation

## Abstract

Chronic inflammation, increased reactivity to self-antigens and incidences of cancer are hallmarks of aging. However, the underlying mechanisms are not well understood. Age-associated alterations in the DNA either due to oxidative damage, defects in DNA repair or epigenetic modifications such as methylation that lead to mutations and changes in the expression of genes are thought to be partially responsible. Here we report that epigenetic modifications in aged DNA also increase its immunogenicity rendering it more reactive to innate immune system cells such as the dendritic cells. We observed increased upregulation of costimulatory molecules as well as enhanced secretion of IFN-α from dendritic cells in response to DNA from aged donors as compared to DNA from young donors when it was delivered intracellularly via Lipofectamine.  Investigations into the mechanisms revealed that DNA from aged subjects is not degraded, neither is it more damaged compared to DNA from young subjects. However, there is significantly decreased global level of methylation suggesting that age-associated hypomethylation of the DNA may be the cause of its increased immunogenicity. Increased immunogenicity of self DNA may thus be another mechanism that may contribute to the increase in age-associated chronic inflammation, autoimmunity and cancer.

## Introduction

Chronic inflammation and increased self reactivity are the hallmarks of aging and are thought to be the underlying cause of the many age-associated diseases such as Alzheimer's; Rheumatoid Arthritis [1]. The mechanisms responsible are not well understood. It is well established that the immune system undergoes significant alterations with age. The consequences of progressive aging of the immune system include an increase in autoimmune phenomena, incidence of cancer, chronic inflammation, and predisposition to infections [2-5]. This suggests a decrease in the protective immune responses to exogenous and infectious agents, and an increase in reactivity to self or endogenous danger signals.  Several factors may be responsible for the increased reactivity to self, including loss of immune tolerance, and progressive age-associated loss of tissue integrity yielding new self-antigens. Another possibility is that advancing age leads to modifications in existing self-antigens [3,4,6] rendering them more immunogenic.

Human DNA is an example of self-antigen that undergoes age-associated genetic and epigenetic alterations [7-11]. It is well documented that aging cells subtly change their patterns of DNA methylation. Overall, cells and tissues become hypomethylated while selected genes become progressively hypermethylated and, potentially, permanently silenced [12,13]. This is believed to be poor prognosis for malignancies [14,15]. A large body of experimental evidence also supports the existence of a relationship between genomic instability, DNA damage and aging [10]. DNA repair functions are severely compromised with age leading to DNA lesions with single and double stranded breaks [15,16]. Moreover, free radicals and reactive oxygen species cause further damage to the DNA and oxidative DNA damage has been implicated in carcinogenesis, ageing [10,11,17] and several age-related degenerative diseases [18]. More recently Rodier et al. [19] have shown that persistent DNA damage in senescent cells induces pro-inflammatory cytokine secretion. It is thus clear that aging leads to alterations in DNA that are implicated in various age-related disorders.

In the present study, we have determined if the age-associated alterations in DNA also affect its immunogenicity. We have tested our hypothesis using a previously established system [20] whereby we deliver human DNA to dendritic cells using transfection agent, Lipofectamine.

## Results

### DNA from aged subjects is more immunogenic than DNA from young subjects

**Figure 1.**
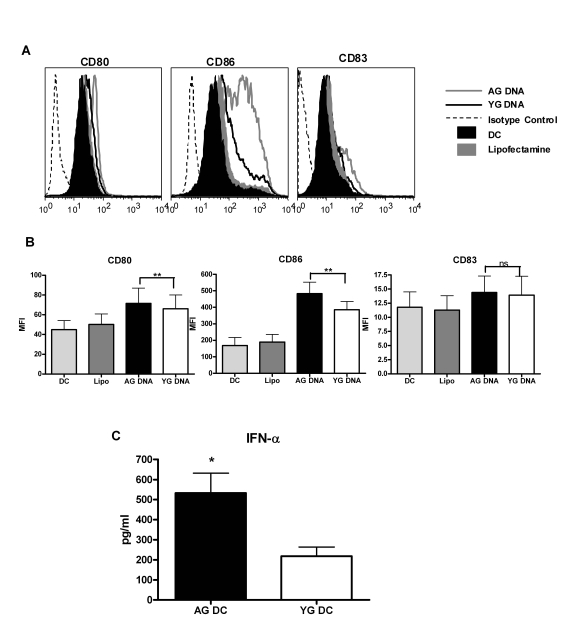
DNA from aged subjects is more immunogenic than DNA from young subjects. (**A**) DCs were activated with aged and young DNA complexed with lipofectamine. The expression of costimulatory molecules CD80 and CD86 and the maturation molecule (CD83) in the unactivated and activated DCs was measured by flow cytometry. Figure is representative of ten such experiments using fifteen separate aged and young DNA. (**B**) Bar graph represents the mean fluorescence intensity of CD80, CD86 and CD83 of the same. (**C**) Supernatants collected from DCs activated with aged and young DNA were assayed for IFN-α using specific ELISA. Bar diagrams depict the concentration of IFN-α secreted by the DCs. Figure is mean + S.E. of thirty separate aged and young DNA tested.

Human DNA is generally inert and does not stimulate dendritic cells (DCs). However, recent studies from our laboratory [20] as well as from others [21-23] suggest that DNA can activate DCs if delivered* in vitro *inside the cell through either transfection or via immune complexes. *In vitro *delivery of DNA mimics the normal physiological conditions where human DNA gains entry into the cells as complexed with proteins or cell debris from dying cells or immune complexes.  Therefore, to investigate our hypothesis, DNA extracted from the blood of aged and young subjects was delivered inside human monocyte derived DCs from healthy young donors using the transfection reagent lipofectamine. The concentration of DNA used was 1 μg/ml. This was found to be the optimal concentration from our previous studies [20] since higher concentrations were toxic while lower concentrations led to insufficient activation of DCs.   Delivery of DNA led to the activation of DCs as evidenced by upregulation of costimulatory markers CD80 and CD86 (Figure 1A and B) and secretion of cytokines IFN-α (Figure 1C). A significantly increased upregulation of CD80 (p=0.008)   and CD86 (p=0.02) was observed in aged DNA (data is of 15 separate aged and young DNA) stimulated DCs compared to DCs stimulated with young DNA (Figure 1A and B). The maturation marker CD83 was not significantly upregulated (p=0.29) in either group.  Cytokine profile shows that there was significantly increased (p=0.003) secretion of IFN-α (data is of 30 separate aged and young DNA, Figure1C) by aged DNA-treated DCs as compared to young DNA-treated DCs. Introduction of lipofectamine alone did not activate DCs.Stimulatory activity of the DNA was lost when delivered without lipofectamine suggesting that exposure to DNA alone does not induce activation of DCs and that this requires its intracellular delivery. These data suggest that aging leads to alterations in DNA that enhance its immunogenicity, and may contribute to age-associated chronic inflammation and autoimmune phenomenon.

### DNA from aged subjects is demethylated compared to DNA from young subjects

**Figure 2.**
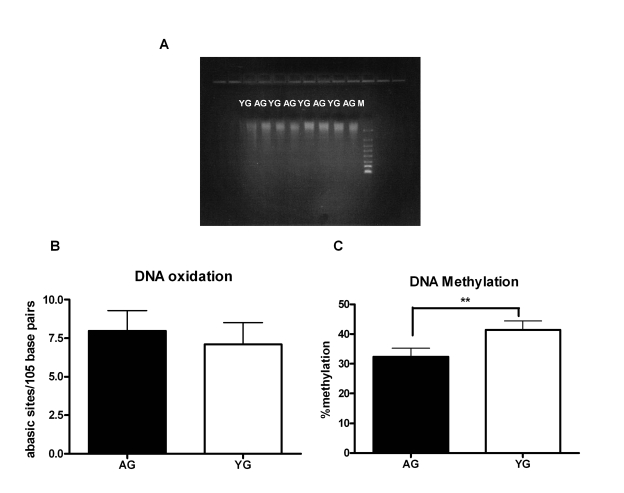
DNA from aged subjects is demethylated compared to DNA from young subjects. (**A**) FlashGel showing the molecular weight of Aged and young DNA. Figure is representative of eight such experiments. (**B**) Bar diagram depicting the damage in DNA from aged and young subjects as determined by ELISA that measures the number of abasic sites per 10^5^ base pairs. Figure is mean + S.E. of twenty five separate aged and young DNA tested. (**C**) Bar diagram depicts the percent of global methylation in aged and young DNA as measured by ELISA. The methylated fraction of DNA is recognized by 5-methylcytosine antibody and quantified through an ELISA-like reaction. Figure is mean + S.E. of twenty four separate aged and young DNA tested.

Next, we investigated the age-associated alterations in the DNA that may be responsible for its increased immunogenicity. Aging is associated with increased apoptosis [24], which may result in shorter fragments of DNA. Running the DNA on gel showed that the size of DNA obtained from both aged and young subjects was comparable (Figure 2A). Accumulation of DNA damage due to either oxidation or inefficient DNA repair mechanism is another characteristic of advancing age [15,16,25]. Therefore, we compared the DNA damage between aged and young DNA [damage of 25 separate aged and young DNA was determined] by measuring the number of abasic sites by ELISA. Indeed, we observed an increase in DNA damage in the DNA from aged subjects relative to DNA from young; however, the difference was not significant (p=0.27, Figure 2B). Age-associated changes in DNA methylation patterns are also a hallmark of aging [8-10,26-29]; both an increase and a decrease DNA methylation have been reported [10,29]. To determine if modifications in methylation are responsible for the immunogenicity of aged DNA, we compared the methylation level ofthe DNA from aged and young subjects using the global DNA methylation ELISA kit (methylation levels of 24 separate aged and young DNA were determined).  In this kit the methylated fraction of DNA is recognized by 5-methylcytosine antibody and quantified through an ELISA-like reaction. There was an approximately ten percent decrease (p=0.001) of DNA methylation in aged as compared to the DNA from young (Figure 2C). This suggests that DNA from aged is hypomethylated, which may result in enhancing its immunogenicity.

### Immunogenicity of mammalian DNA correlates inversely with DNA methylation

**Figure 3.**
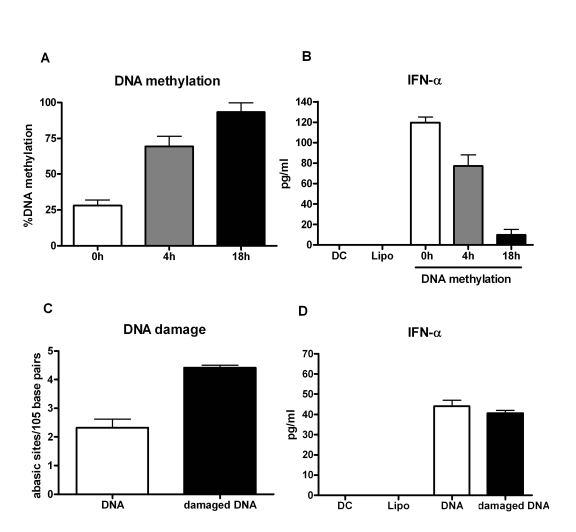
Immunogenicity of mammalian DNA correlates inversely with DNA methylation. (**A**) DNA was methylated using a methyl transferase enzyme and percent global methylation was measured by ELISA. Bar diagram shows the percent of global methylation at 0h, 4h and 16h after the reaction. Figure is mean + S.E. of five separate DNA tested.  (**B**) The immunogenicity of the methylated DNA was determined by measuring the IFN-α secretion by DCs.  Bar diagram shows the level of IFN-α secreted by DCs in response to the DNA. Figure is mean + S.E. of five separate DNA tested. (**C**) PBMCs were treated with hydrogen peroxide to induce DNA damage. Damaged DNA was extracted and the extent of damage was determined by ELISA. Bar diagram shows the level of DNA damage before and after treatment. Figure is mean + S.E. of five separate DNA tested (**D**) The immunogenicity of the H_2_O_2_ damaged DNA was determined by measuring the IFN-α secretion by DCs. Bar diagram shows the level of IFN-α secreted by DCs in response to the DNA. Figure is mean + S.E. of five separate DNA tested.

To further confirm if hypomethylation of aged DNA influencesthe immunogenicity of the DNA, we *in vitro* methylated the DNA from aged subjects using a methyl transferase enzyme. The percent of DNA methylation correlated with time with increased methylation being observed at 18h compared to 4h of the reaction (Figure 3A).The experiment was repeated with five separate DNAs. In two experiments commercially obtained DNA from Jurkat and Hela cell lines were used for methylation. This was done to confirm that the results obtained are not an artifact of our purification process.

Furthermore, intracellular delivery of this methylated DNA into DCs resulted in decrease in IFN-α secretion compared to untreated DNA control (Figure 3B). The decrease in IFN-α production correlated with increase in DNA methylation. These data clearly demonstrate that epigenetic changes in the DNA from aged subjects' leads to decreased DNA methylation resulting in an enhanced immunogenicity of the DNA.

Since our results demonstrated a small but insignificant increased damage in the DNA from aged, we investigated if this damage also affected the immunogenicity of the DNA.  PBMCs from young subjects were exposed to hyderogen peroxide to induce DNA damage. This treatment led to an increased DNA damage as shown in Figure 3C; however, intracellular delivery of this damaged DNA into DCs did not result in increased activation compared to undamaged DNA from the same individual (Figure 3D). This suggests that DNA damage does not alter its immunogenicity further confirming that hypomethylation of the DNA is responsible for its increased immunogenicity.

## Discussion

Decreases in global level of methylation along with a concomitant increase in promoter methylation are the hallmark of age-associated epigenetic changes [10,29].

These age-associated epigenetic changes are thought to play a key role in the development of cancer and autoimmune phenomena through modification of gene expression. Causes for age-related methylation changes remain unknown.

Accumulating studies have indicated a potential role of DNAhypomethylation in the pathogenesis of autoimmune diseases [30-36]. Earlier *in vivo *studies have shown potentiation of autoimmunity in mice treated with hypomethylating agents such as5-azacytidine, procainamide and hydralazine [31,34].Others studies described that DNA hypomethylation is essential for apoptotic DNA to induceSLE-like autoimmune disease in non-susceptible mice [35]. Changes in human DNA methylation patterns are also an important feature of cancer development and progression [14,33,36,37]. Alterations of DNA methylation are one of the most consistentepigenetic changes in human cancers [37]. Human cancers generallyshow global DNA hypomethylation accompanied by region-specifichypermethylation a pattern similar to aging. Studies have shown hypomethylation of squamous cell carcinomas in White men was associated with shorter survival from the disease [38]. A potential role of DNA hypomethylation in other conditions such as atherosclerosis and autoimmune diseases [e.g., multiple sclerosis and lupus] is also being recognized [39,40].

As is evident abnormal DNA methylation plays a very important role in various pathologic states, such as leukemia and autoimmunity. The underlying mechanisms are however not fully delineated so far. Our data suggests that along with regulating the transcription of various genes these methylation changes also render the DNA to be more immunogenic. The immune system is normally protected from exposure to self dsDNA during apoptosis due to the rapid engulfment of apoptotic cells, and the abundance of extra- and intracellular DNases [41,42]. However, phagocytic cells may be exposed to cellular DNA following tissue necrosis, inflammation, or viral infection. Defective clearance of apoptotic cells would also result in an accumulation of late phase apoptotic cells. Previous study from our laboratory in humans [43] and a recent study [44] in mice suggest that apoptotic cell clearance is decreased with age. This may result in release of DNA from apoptotic cells due to secondary necrosis. Such DNA in aging would be much more immunogenic since it is hypomethylated and would lead to maturation of dendritic cells and increased reactivity to self resulting in slow loss of peripheral self tolerance. The increased immunogenicity of hypomethylated DNA may thus be one of the mechanisms that contribute to the development of autoreactivity, cancer and chronic inflammation associated with aging.

Future studies of pivotal interest would be the identification of the receptor and signaling pathways involved in the recognition of this hypomethylated DNA. Earlier studies with intracellular DNA delivered via transfection reagents have shown that DNA signals through non-TLR receptors [21-23]. Our own study [20] also found that the DNA was localized in the cytosol and was not accessible to intracellular TLRs in the endosomes. The nucleic acid sensing TLR3 and TLR8 are found in the endosomes [45]. The two other known nucleic acid sensing TLRs, TLRs 7 and 9 are not expressed in human monocyte derived DCs and are also present in the endosomes [46]. Identification of the receptor would also provide novel target for therapy of autoimmune diseases.

## Materials and method

Blood donors. Blood was collected from healthy elderly (age 65-90 years) and young volunteer (age 20-35 years) donors. Elderly subjects belong to middle-class socio-economic status and are living independently. A week prior to the study, they were asked to discontinue any vitamins, minerals and antioxidants that they may have been taking. The Institutional Review Board of the University of California, Irvine, approved this study.

Preparation of human monocyte derived dendritic cells. DCs were prepared essentially as described [43]. Briefly, peripheral blood mononuclear cells (PBMCs) were separated by Ficoll-Hypaque density gradient centrifugation. Monocytes were purified from the PBMCs by positiveselection with anti-CD14 microbeads (Stemcell Sep, Vancouver, BC). The purity of the isolatedCD14^+^ monocytes was >90% as determined by flow cytometry.For the induction of DC differentiation, purified CD14^+ ^monocytes were cultured in a humidified atmosphere of 5%CO_2_ at 37°C in RPMI 1640 supplemented with10%FBS, 1 mMglutamine, 100 U/ml penicillin, 100 μg/ml streptomycin,50 ng/ml human rGM-CSF (PeproTech, Rocky Hill, NJ), and 10 ng/ml human rIL-4(Peprotech). Half of the medium was replaced every2 days and DCs (CD14^-^HLA-DR^+^CD11c^+^cells) were collected after 6 days. The purity of the DC was >95% as determined by the expression of CD14, CD11c and HLA-DR.

Self DNA Preparation. DNA was isolated from the blood of aged and young subjects using Qiamp DNA Blood Midi Kit from Qiagen (Valencia, CA). RNase was added to remove any contaminating RNA. Purity and yield of DNA was measured by UV spectrophotometer. Preparations with 260/280 ratio above 1.9 were used in all experiments. DNA obtained was free of endotoxin contamination as determined by Limulus amoebocytelysate (LAL) assay (Lonza Inc, Allendale, NJ).

DC activation. Transfection reagent Lipofectamine 2000 (Invitrogen, Carlsbad, CA) was used to deliver self DNA to DCs. DNA was mixed with lipofectamine (lipo) in 100 μl of Opti-MEM for 20 minutes at room temperature, according to the protocol recommended by the manufacturer and added to 4×10^5^ DCs in 300 μl of complete medium. Final concentration of the DNA was 1μg/ml. Cell viability was unaffected by this treatment. Unstimulated and Lipofectamine-stimulated DCs were used as controls. After 24 hours, cells were harvested and stained for surface markers CD80, CD86 and CD83, using directly conjugated antibodies and isotype controls (BD Pharmingen, San Jose, CA). 10,000 CD11c^+^HLA^-^DR^+^ cells per condition were acquired using a FACSCalibur (BDPharmingen, San Jose, California).  Analysis was performed using the Flow jo software (Treestar Inc, Ashland, OR).

Cytokine, IFN-α  in the supernatants was measured by verikine IFN-α measuring kit (PBL Biomedicals, Piscatway, NJ) as per the manufacturer's protocol.

Quantification of DNA methylation. Global methylation of DNA from aged and young subjects was determined using the *Methylamp™ Global DNA Methylation Quantification Kit from Epigentek (Brooklyn, NY), as per the manufacturer's protocol. In this kit the* methylated fraction of DNA is recognized by 5-methylcytosine antibody and quantified through an ELISA-like reaction.

Methylation of DNA. 1μg of DNA was methylated in GC Reaction Buffer containing 60 units of GpC Methyltransferase (M.CviPI) and 160 μM S-adenosylmethionine from New England Biolabs (Ipswich, MA) at 37^o^C for either 4 or 18 hours.  The GC Methyltransferase methylates all cytosine residues within the double stranded recognition sequence of 5'..GC..3'. Percent methylation was determined using the global DNA methylation kit.

Induction of DNA damage. PBMCs from young donors were treated with 10uM hydrogen peroxide for 1 h at 37^o^C. DNA was extracted as described from both treated and untreated PBMCs. Damage was assessed using the DNA damage quantification kit.

Quantification of DNA damage. DNA damage from aged and young subjects was quantified using DNA damage Quantification kit from BioVision (Mountain View, CA) following the manufacturer's protocol. This kit determines the number of abasic sites in purified DNA samples utilizes the ARP (Aldehyde Reactive Probe) reagent that reacts specifically with an aldehyde group, which is the open ring form of the Apurinic/apyrimidinic ( AP) sites.

Statistics. Statistical analysis was performed using GraphPad Prism™ 4.00 software (GraphPad Software, San Diego, USA). Unpaired data were analyzed with the Mann-Whitney test. Wilcoxon signed ranked test was used for paired analyses. Statistical significance was acknowledged when the *P*-value was <0.05.
